# Experiences of Latinx Individuals Hospitalized for COVID-19: A Qualitative Study

**DOI:** 10.1001/jamanetworkopen.2021.0684

**Published:** 2021-03-11

**Authors:** Lilia Cervantes, Marlene Martin, Maria G. Frank, Julia F. Farfan, Mark Kearns, Luis A. Rubio, Allison Tong, Andrea Matus Gonzalez, Claudia Camacho, Adriana Collings, William Mundo, Neil R. Powe, Alicia Fernandez

**Affiliations:** 1 Division of Medicine, Denver Health, Denver, Colorado; 2 Office of Research, Denver Health, Denver, Colorado; 3 Department of Medicine, University of Colorado, Aurora; 4 Zuckerberg San Francisco General Hospital, Department of Medicine, University of California San Francisco, San Francisco; 5 University of Illinois College of Medicine, Chicago; 6 Sydney School of Public Health, University of Sydney, Sydney, Australia; 7 Centre for Kidney Research, Children’s Hospital at Westmead, Westmead, New South Wales, Australia

## Abstract

**Importance:**

Latinx individuals, particularly immigrants, are at higher risk than non-Latinx White individuals of contracting and dying from coronavirus disease 2019 (COVID-19). Little is known about Latinx experiences with COVID-19 infection and treatment.

**Objective:**

To describe the experiences of Latinx individuals who were hospitalized with and survived COVID-19.

**Design, Setting, and Participants:**

The qualitative study used semistructured phone interviews of 60 Latinx adults who survived a COVID-19 hospitalization in public hospitals in San Francisco, California, and Denver, Colorado, from March 2020 to July 2020. Transcripts were analyzed using qualitative thematic analysis. Data analysis was conducted from May 2020 to September 2020.

**Main Outcomes and Measures:**

Themes and subthemes that reflected patient experiences.

**Results:**

Sixty people (24 women and 36 men; mean [SD] age, 48 [12] years) participated. All lived in low-income areas, 47 participants (78%) had more than 4 people in the home, and most (44 participants [73%]) were essential workers. Four participants (9%) could work from home, 12 (20%) had paid sick leave, and 21 (35%) lost their job because of COVID-19. We identified 5 themes (and subthemes) with public health and clinical care implications: COVID-19 was a distant and secondary threat (invincibility, misinformation and disbelief, ingrained social norms); COVID-19 was a compounder of disadvantage (fear of unemployment and eviction, lack of safeguards for undocumented immigrants, inability to protect self from COVID-19, and high-density housing); reluctance to seek medical care (worry about health care costs, concerned about ability to access care if uninsured or undocumented, undocumented immigrants fear deportation); health care system interactions (social isolation and change in hospital procedures, appreciation for clinicians and language access, and discharge with insufficient resources or clinical information); and faith and community resiliency (spirituality, Latinx COVID-19 advocates).

**Conclusions and Relevance:**

In interviews, Latinx patients with COVID-19 who survived hospitalization described initial disease misinformation and economic and immigration fears as having driven exposure and delays in presentation. To confront COVID-19 as a compounder of social disadvantage, public health authorities should mitigate COVID-19–related misinformation, immigration fears, and challenges to health care access, as well as create policies that provide work protection and address economic disadvantages.

## Key Points

**Question:**

Can experiences of Latinx adults hospitalized with coronavirus disease 2019 (COVID-19) inform improvements to public health and health care?

**Findings:**

In this qualitative study of 60 Latinx adults, participants reported COVID-19 misinformation, felt COVID-19 compounded existing social disadvantage, and risked infection because of the need to work. Participants hesitated to seek hospital care because of immigration and economic concerns.

**Meaning:**

These findings suggest that to contain community spread and reduce unnecessary morbidity, immigration, employment, and economic distress must be addressed through tailored public health messaging and public policy interventions that improve economic conditions.

## Teaser

This qualitative study describes the experiences of Latinx adults who were hospitalized for and survived coronavirus disease 2019 (COVID-19) in 2 major US cities.

## Introduction

Coronavirus disease 2019 (COVID-19) has magnified preexisting health and social inequities stemming from long-standing poverty, structural racism, and immigration status. As a result, certain racial and ethnic groups, including Latinx individuals (who are a part of the largest ethnic minority in the US at 60 million), are overrepresented among COVID-19 infections.^1,2,3,4,5,6,7^ Current data show that compared with White individuals, Latinx individuals are more likely to become infected, hospitalized, and die from COVID-19.^8,9,10,11,12,13,14^ In addition, Latinx individuals have had some of the highest rates of excess mortality compared with other racial and ethnic groups and have not been shown to benefit from shelter-in-place policies.^14^

Multiple factors drive excess COVID-19 risk among Latinx individuals.^15^ Much of the excess risk in Latinx communities is concentrated among immigrants.^16^ Compared with other racial and ethnic groups, Latinx immigrants are more likely to work in low-wage, service industries and be uninsured.^17,18,19,20,21,22^ A recent analysis of individuals with COVID-19 found that compared with non-Latinx groups, Latinx individuals were more like to report working while ill, exposure to someone with COVID-19 in the household, and having more persons in the household.^13^ These circumstances have contributed to an excess burden of COVID-19 morbidity and mortality in the Latinx community.^13,14^

In this study we describe Latinx patient perspectives on COVID-19 before, during, and after hospitalization in 2 cities where Latinx people are disproportionately affected. Learning from the experiences of Latinx people who had been hospitalized with COVID-19 can inform local and national interventions to reduce avoidable COVID-19 infections and decrease COVID-19 morbidity and mortality in the Latinx community.

## Methods

### Study Design, Participants, and Settings

We conducted semistructured interviews with Latinx adults (age ≥18 years) who had been hospitalized for COVID-19 in public hospitals located in Denver, Colorado, and San Francisco, California, between March and July 2020. Both cities reported excess COVID-19 cases among Latinx individuals compared with their numbers in the overall population (54% vs 32% in Denver and 51% vs 15% in San Francisco).^23,24^ We identified participants via a data query that provided the contact information for individuals who self-identified as Latinx and had been hospitalized for COVID-19, and had an interviewer call them. Purposive sampling captured a diverse sample in terms of gender and city. Participants provided informed consent that included permission to publish deidentified quotations. The institutional review boards of the University of Colorado, Denver, and the University of California, San Francisco, approved this study. We report our study using the Consolidated Criteria for Reporting Qualitative Research (COREQ) reporting guideline.

### Data Collection

Six authors conducted semistructured interviews in English or Spanish by telephone. The interview guide (eTable 1 in the Supplement) was based on a literature review of race disparities and the COVID-19 pandemic, with a particular focus on Latinx communities.^1,2,21,25,26,27,28^ Interviews were audio recorded and transcribed verbatim, and were conducted until thematic data saturation, defined as when a thorough understanding of the perspectives of Latinx respondents were obtained with few or no new concepts emerging in subsequent data collection.^29,30^

### Data Analysis

Interview transcripts were imported into HyperRESEARCH^31^ version 4.0.1 (ResearchWare Inc). Five authors (L.C., M.M., M.F., J.F.F., and A.F.) identified initial themes. Coding and analysis was performed according to the principles of grounded theory and thematic analysis.^29,30^ Authors A.T. and A.M.G. performed line-by-line coding to inductively identify initial concepts, then grouped similar concepts into themes and subthemes, and identified conceptual links among themes. They reached consensus on themes with authors L.C., M.M., M.F., and A.F. Investigator triangulation ensured that the themes reflected the full range and depth of the data.

## Results

Sixty individuals (36 [60%] men; mean [SD] age, 48 [12] years) participated. All lived in low-income areas, 47 participants (78%) had more than 4 people in their home, and 44 participants (73%) were classified as essential workers.^32,33^ Four participants (9%) could work from home, 12 (20%) received sick leave, and 21 (35%) lost their job due to COVID-19. Fifty-four interviews (90%) were conducted in Spanish. Participants were hospitalized for a mean (SD) 8 (10) days and 17 participants (28%) required treatment in the intensive care unit (Table 1; eTable 2 in the Supplement). Of those called to participate, 77 (78%) agreed. The most common reasons for declining were fear of sharing personal information, lack of time, and fatigue. Mean (SD) interview duration was 42 (12) minutes.

**Table 1.** Characteristics of Adult Latinx Survivors Hospitalized for COVID-19

Characteristic	Total, No. (%) (N = 60)
Age, mean (SD), y	48 (12)
Female	24 (40)
Location	
Denver Health	30 (50)
Zuckerberg San Francisco General Hospital	30 (50)
Preferred interview in Spanish	54 (90)
Socioeconomic	
Below high school education	38 (63)
Household income, $	
<25 000	12 (38)
25 000-34 999	12 (20)
35 000-49 999	9 (15)
50 000-74 999	6 (10)
Married	23 (38)
Residence in low-income area	60 (100)
>4 people in home	47 (78)
>1 bathroom in home	23 (38)
>2 bedrooms in home	44 (73)
>1 person with COVID-19 at home	50 (83)
Type of work	
Essential work^a^	44 (73)
Critical trades (construction, electrician, plumbers, etc)	20 (33)
Agriculture/food production (restaurant, animal and crop production)	12 (20)
Critical retail (grocery stores, hardware stores, mechanics)	6 (10)
Transportation	2 (3)
Health care providers (certified nurse and medical assistants)	3 (5)
Childcare	1 (2)
Other	2 (3)
Unemployed	14 (23)
Employment characteristics (n = 46)	
Ability to work from home	4 (9)
Received sick leave	12 (26)
Use of personal protective equipment at work	32 (70)
Employer informed of COVID-19 diagnosis	37 (80)
Lost job due to COVID-19	21 (46)
Type of transportation	
Public	20 (33)
Private	40 (67)
Insurance type	
DFAP/Healthy San Francisco^b^	18 (30)
Emergency Medicaid	18 (30)
Medicaid	19 (32)
Medicare	2 (3)
Commercial	2 (3)
Clinical	
Symptoms on admission	
Cough	47 (78)
Shortness of breath	44 (73)
Abdominal pain	12 (20)
Diarrhea	18 (30)
Myalgia	26 (43)
Dysgeusia	4 (7)
Anosmia	8 (13)
Flu shot received in the past year	19 (32)
BMI, mean (SD)	
<30	27 (45)
30-34.9	18 (30)
≥35	15 (25)
Comorbidities	
Diabetes	23 (38)
Hypertension	18 (30)
Cardiovascular disease	2 (7)
Chronic lung disease	6 (2)
Hospital course	
Hospital length of stay, mean (SD) d	8 (10)
Intensive care unit stay	17 (28)
Intensive care unit, length of stay, mean (SD) d	17 (28)
Intubated	10 (17)
ARDS	13 (22)
Acute kidney failure^c^	3 (5)
Acute liver injury^d^	1 (2)
Co-infection with bacterial pneumonia	5 (8)
Disposition	
Home	48 (80)
Hotel	10 (17)
Other	2 (3)
Discharged with oxygen	10 (17)

Abbreviations: ARDS, acute respiratory distress syndrome; BMI, body mass index (calculated as weight in kilograms divided by height in meters squared); COVID-19, coronavirus disease 2019; DFAP, Denver Health Financial Assistance Program.

^a^ Essential workers are considered essential at the state level, which required they continue to show up to work during the different phases of the restrictions states implemented during the COVID-19 pandemic.^32,33^

^b^ DFAP provides hospital coverage for undocumented Denver residents; Healthy San Francisco provides health coverage for undocumented San Francisco residents.

^c^ Acute kidney injury defined as increase in serum creatinine by 0.3mg/dL or more (>26.5 μmol/L; to convert creatinine to micromoles per liter, multiply by 88.4) within 48 hours or increase in serum creatinine up to 1.5 times or more baseline within prior 7 days compared with preceding 1 year of data in acute medical records.

^d^ Acute liver injury defined as an elevation of aspartate aminotransferase or alanine aminotransferase of more than 15 times the upper limit of normal.

We identified 5 themes and subthemes with public health and clinical care implications, which are provided in Table 2 with illustrative quotations. Conceptual links are shown in the eFigure in the Supplement.

**Table 2.** Themes, Subthemes, and Illustrative Quotes From Adult Latinx Survivors Hospitalized for COVID-19

**Theme: COVID-19 was a distant and secondary threat**	
Invincibility	“I would hear COVID-19 information and think I’d never get it. It isn’t until you get COVID-19 that you realize the truth. I was very sick and now I’m too scared to leave my house.” (Participant 26)
	“Unfortunately, we Latinos think that illnesses come and go, and that they will not affect us, and if we are affected, that we don’t need to give them attention. That’s the problem, we don’t take it seriously.” (Participant 41)
Misinformation and disbelief	“The men go to the streets and work then they go to the grocery store and their kids run around. I think they don’t protect themselves because they don’t know. There is no information.” (Participant 3)
	“I obtained most of my information from Facebook … I didn’t understand how to protect myself at first.” (Participant 17)
	“I kept hearing we needed to hold our breath and so that is what I did … and then I got sick.” (Participant 54)
Ingrained social norms	“We became sick at the same time because we were all together. My sister-in-law was pregnant and had COVID-19. We all got together, and she was with us. She didn’t know that she had COVID-19 because she didn’t have symptoms.” (Participant 12)
	“I was careful for weeks and then I had a gathering at my house and I invited a lot of people and that is how I think I got sick.” (Participant 47)
**Theme: COVID-19 was a compounder of disadvantage**	
Fear of unemployment and eviction	“I feared not fully recovering … I wasn’t sure if my boss would let me keep my job … I knew that I was dispensable and they could fill my position … I’d understand if they fired me for being unable to work because of COVID-19.” (Participant 10)
	“If we get evicted because we can’t pay rent, that’s when we all help each other, and then that’s when families come together and live together. You live with friends or families, but then that’s how we also all expose each other.” (Participant 46)
	“My fear is that they close all the restaurants and then there is no work, and no way to pay the rent or bills. This worries me because we live day to day.” (Participant 47)
Lack of safeguards for undocumented immigrants	“I was worried because my husband could be unemployed and then how would we pay our bills? Being an immigrant, we are at a disadvantage. We know that we won’t have the same support.” (Participant 5)
	“I am scared of not having a job because being undocumented and sick, there isn’t help. One has to work. This is the main worry that we all have.” (Participant 13)
Inability to protect self from COVID-19	“I had to leave the house daily to work … they gave us gloves but no mask … I worked cleaning apartments and well, they only gave us gloves.” (Participant 13)
	“I think I got COVID-19 because I was using public transportation to get to work … when I would get on the bus, there were lots of people without face masks or gloves and it didn’t look like they had their own hand sanitizer either.” (Participant 46)
High-density housing	“We Mexicans may have 2 or 3 families in the same home, with kids in the same house, and well … that is very dangerous because the infection is very contagious.” (Participant 3)
	“We have no space. If one room in the house gets sick, it goes to the next room … that is what most worried me. Even if we followed the rules, we can’t distance in the home. It’s complicated when you can’t move in your home.” (Participant 59)
**Theme: reluctance to seek medical care**	
Worry about health care costs	“I was worried about getting sick and needing to go the hospital, because then there are bills, and it is not easy to pay those bills. It becomes very difficult for someone who isn’t from this country. Every day while I was in the hospital, I thought about the bill. Believe me, this was so distressing, knowing that each extra day would cost more.” (Participant 18)
	“I kept telling myself, to force myself to stay home, to bare the breathlessness, but once I couldn’t breathe anymore, there was no other option.” (Participant 19)
	“I did not have health insurance. I was scared of the bill, but I had to go because I couldn’t handle the fever.” (Participant 55)
Concerned about ability to access care if uninsured or undocumented	“As an undocumented Latino, the truth is that one wonders, when one gets this illness and is unemployed and without money, how will one pay for this? And if we can’t pay, can we receive care?” (Participant 18)
	“When one is hospitalized, all one can think about is the bills and well, one does not have support because of immigration status—because not all of us have documents.” (Participant 18)
Undocumented immigrants fear deportation	“People go very scared, and I understand because I was in the same situation, not knowing if you’ll receive care, not knowing the steps, not knowing if your information will be shared.” (Participant 3)
	“We fear going to the doctor, hospital, or clinic. We are fearful because we think that they will share our information and immigration officials will take you, incarcerate you, and deport you.” (Participant 10)
	“I am undocumented and in this country without papers. I have been here for 28 years and so you know, one worries that the hospital will call ICE, and that they will come and take us. I don’t leave my house and worry so much.” (Participant 44)
**Theme: health care system interactions**	
Social isolation and change in hospital procedures	“I was scared of going to the hospital because once you’re there, you can’t have any visitors, you are alone and if something happens to you, your family can’t come visit you. I was very scared that I would die there.” (Participant 42)
	“Family cannot come in and see you, not even if you’re in your last days, and so that it was caused me the most distress … to not see my children again.” (Participant 54)
Appreciation for clinicians and language access	“I was very scared and lonely but I called my family using facetime and the nurses were wonderful. They came to the room and I know they couldn’t stay long because everyone is scared of getting this, but they did a great job taking care of me.” (Participant 2)
	“My appreciation for the care is infinite. Infinite because everyone was always attentive, and my wife and I are so grateful … the doctors, the nurses, the housekeepers, these are divine, extraordinary people.” (Participant 3)
	“The clinicians were so kind, they gave me motivation and when you are sick, you need motivation, because sometimes when you are sick you have to depend on others and well, the clinicians lifted your spirits by giving you their hand.” (Participant 18)
Discharge with insufficient resources or clinical information	“I don’t have health insurance and so I had to pay for the oxygen when I was discharged. I used my credit card. I had to pay before I left the hospital … all I wanted was to leave and even though it meant I would have to work more to pay for the oxygen, well I just wanted to leave.” (Participant 13)
	“They didn’t inform me well about the treatment, about what the medication would do. I didn’t understand the side effects to make a decision about taking or not taking the medication and being in the study. They didn’t tell me what would happen if the study medication didn’t work and I ended up back in the hospital.” (Participant 23)
**Theme: faith and community resiliency**	
Spirituality	“I put myself in God’s hand and I said, ‘Dear God, I know that you have my family in your hands,’ … we must all have faith in God that this is all going to pass and that someday, everything will be normal again.” (Participant 8)
	“I just felt like God is in control. When one is a believer, one believes that everything is in God’s hands. If I came back to my family or I died, it would be God’s will.” (Participant 17)
	“I had a lot of faith in God, faith that the illness would leave me and that I would be survive this. It’s not good to think about bad or sad things … but if one has faith in God, then one can survive this.” (Participant 45)
Latinx COVID-19 as advocates	“Our community believes COVID-19 won’t affect us … I now urge people to listen … I say, ‘Take care of yourselves, use the face mask, maintain distance, wash all the things that you buy like fruit, and don’t have gatherings.’” (Participant 41)
	“The Latino community needs to know that we are the ones getting sick and dying … that it’s not a joke … that we need to protect our families, use a mask, and wash our hands.” (Participant 36)
	“The truth is that I didn’t take this seriously. Even though I was hearing about it on the news … people need to learn to maintain their 6 feet distance, use the face masks, wash their hands, use hand sanitizer, all of these measures are necessary. Sometimes, people don’t understand until someone ends up sick and in bed.” (Participant 60)

Abbreviation: COVID-19, coronavirus disease 2019.

### COVID-19 Was a Distant and Secondary Threat

#### Invincibility

Some participants felt they would not contract COVID-19, and if infected they would not become ill (“I initially ignored it and did not use a mask because I thought I wouldn’t get sick.”). Many felt the media was “overexaggerating,” and dismissed preventive recommendations. One participant highlighted the indifference among his coworkers, saying, “We’d make fun of COVID. If one of us coughed, we’d say, ‘you have COVID,’ and laugh.” They regarded some measures, including workplaces closing, to be too extreme: “When they told us to stop working at the restaurant I asked, ‘Why, if we’re fine?”

#### Misinformation and Disbelief

Many participants relied on social media for COVID-19 recommendations and described a lack of information and misinformation. Some recounted that before they became ill, they thought COVID-19 “was a bunch of lies.” Others were suspicious of the government. “I thought the government invented COVID,” said a participant. “It wasn’t until I got sick that my family and I believed it.” A few believed that COVID-19 was aimed at identifying undocumented individuals (“There is lack of information and understanding about COVID. Some of us see it as a tactic for the government to access our documentation status and deport us.”).

#### Ingrained Social Norms

Some participants found it difficult to physically distance because of cultural norms. “As a community, we demonstrate affection,” said a participant. “When we see someone we know, we give a firm handshake, a strong hug, and some even get a kiss.” Participants described gatherings during shelter in place: “Latinos get together for graduations and birthdays and that’s where we get sick. …I understand now because I ignored it and that’s how I got sick.”

### COVID-19 Was a Compounder of Disadvantage

#### Fear of Unemployment and Eviction

Participants were terrified they would become unemployed because of business closures or a COVID-19 diagnosis. “I wasn’t sure if we would all be fired [because of shelter in place],” said a participant. “After getting sick, my main concern still was being fired.” Some participants supported relatives abroad and worried about their family’s well-being (“How will I support my family in Mexico if I can’t send money?”). Even with COVID-19 symptoms, individuals felt a need to work (“I worked on days that I was feeling terrible, because I had no money and I knew more difficult times were coming.”). Participants were also concerned over losing their home (“I worried that once discharged from the hospital, I wouldn’t have a home, and the landlord would tell me that I couldn’t live there anymore because I couldn’t pay the rent.”).

#### Lack of Safeguards for Undocumented Immigrants

Undocumented participants reported anxiety because of their lack of resources because of their immigration status. “I’ve been worried about unemployment, because in this country, if you don’t have rent money, you are thrown into the streets,” said a participant. “You don’t have food either … as an undocumented person, there are no benefits.” Undocumented immigrants described feeling dehumanized and unprotected (“We are just surviving … in this country, I have nowhere I can go for help.”).

#### Inability to Protect Self From COVID-19

Because of financial constraints, many participants continued working despite shelter in place. “Regardless of the risk, we have to work to make money,” said a participant. “Other people can work from home but our jobs don’t allow us to.” Those who continued working found it difficult to protect themselves because of their occupation. One participant said, “We couldn’t protect ourselves because our work requires heavy lifting and we are breathing hard, and the mask doesn’t help.”

#### High-Density Housing

Many participants live in multigenerational housing and were anxious about infecting their families. “I was worried about my family,” said a participant. “I would arrive home from work and hope I was not sick. I didn’t want to spread it to my whole family.” Others lived in small, crowded settings where they found it impossible to practice physical distancing (“The rent is expensive. We do not have the necessary space to safely isolate from others … there are 6 or 7 people living in each bedroom.”). They had to trust that others in the same home were taking precautions (“We all have to work. We don’t know who has COVID.”).

### Reluctance to Seek Medical Care

#### Worry About Health Care Costs

Many avoided seeking health care because of concerns about cost—“The cost was the main reason I didn’t want to go to the hospital. If I die, how will my family pay for it?” Participants waited until symptoms were advanced. “I couldn’t take the severe pain in my eyes,” said one. “I didn’t want to go to the hospital because I was afraid of the bills … I have no benefits.”

#### Concerned About Ability to Access Care if Uninsured or Undocumented

Uninsured participants were uncertain they would receive care even in the safety net. One participant said, “When I got sick, I feared going to the hospital because I was scared that they wouldn’t provide me care.” Some felt that because of being undocumented, they would be withheld care (“When I got sick, I worried about being an immigrant, and not receiving medical care.”).

#### Undocumented Immigrants Fear Deportation

Patients were afraid that if hospitalized, their immigration status would be assessed and reported. As one participant said, “If I don't have my legal documents, they can report that information.” Individuals feared being deported if they were hospitalized (“When they realize that you do not have papers after arriving at the hospital, they can deport you.”).

### Health Care System Interactions

#### Social Isolation and Change in Hospital Procedures

While hospitalized, participants felt loneliness and wanted more direct contact with their clinicians. “I didn’t understand at first,” said one participant. “They would come in but not touch me … they were coming in with lots of precautions … it hurt my feelings … its dehumanizing.” Individuals described being treated differently because of COVID-19. “They wouldn’t take me to the bathroom when I needed to go,” said a participant. “They wouldn’t give me physical therapy even though I needed it.” Participants with previous hospitalizations acknowledged that COVID-19 hospital care was different (“They never cleaned my room during the weeks that I was there, they never took out the trash. This time was different.”). Restrictive visitor policies led to loneliness for those that had family in the area. Others expressed anxiety about being sick in a country far from family (“What am I going to do if I get very sick because my family is in a different country? I’m scared.”).

#### Appreciation for Clinicians and Language Access

Many participants reported relief and gratitude for clinicians that motivated them to cope with COVID-19. “The nurses and doctors, they encouraged me,” said a participant. “They would say, ‘Have hope because you will get over this.’” Participants also described readily available language services (“The doctors were always using interpreters and thank God that many of the nurses spoke Spanish.”). Many expressed preferences for Spanish-speaking clinicians (“I think it is important that nurses speak Spanish because we spend a lot of time with them.”). And participants expressed gratitude for their care (“They took excellent care of me…I am so grateful.”).

#### Discharge With Insufficient Resources or Clinical Information

On discharge, some participants described leaving without follow up treatment and care, including oxygen and physical therapy, because they lacked health benefits. “I had low oxygen and when they took it off, it went below low,” said a participant. “They could not offer oxygen at time of discharge because I couldn’t pay for it.” After discharge, many worried about how long they were supposed to continue isolation, and wondered if they could become re-infected (“Once I was extubated, they didn’t tell me that I had already passed the [isolation time] and that I wouldn’t be contagious anymore.”).

### Faith and Community Resiliency

#### Spirituality

Some participants described relying on their faith and prayed that they would not die (“I would say, my dear God, take this illness away from me.”). Many prayed for their families because they did not want them to be sick. They also prayed that if they died, their families would be okay (“Dear God, if I die, what will happen to my child? It’s God’s will as he watches us.”).

#### Latinx COVID-19 Advocates

Worried that COVID-19 would continue spreading in the Latinx community, participants urged their friends and family to protect themselves (“I tell all of my family that they need to understand COVID-19 because it is awful.”). Many felt that the Latinx community was more likely to believe that COVID-19 is real if they received information from a Latinx community member. Said one participant, “It’s important to tell people that you were sick because then they can believe … for example, out of every 100, there may be 10 that know someone with COVID-19, and they can say, ‘Yes, this virus is real because I know someone who is sick,’ … Sometimes the television commercials show people from a different race or language and we think, ‘Oh, that’s not real.’ But if we see people that we know with COVID, then we will believe what is happening.”

## Discussion

This report on the experiences of Latinx adults who survived COVID-19 before, during, and after hospitalization identified multiple themes with public health implications. Common themes included the prevalence of COVID-19 misinformation, COVID-19 as compounding socioeconomic disadvantage, and a reluctance to seek medical care. We also identified themes with implications for health care systems including experiences of social isolation during hospitalization, difficulty with discharge planning, and the role of faith and community in recovery. These findings have implications for COVID-19 control both within and outside the Latinx community, because Latinx individuals have the highest rates of employment in jobs that require essential workers, in grocery stores and restaurants, general merchandise stores, pharmacies, and other businesses where they come into contact with the community at large. Our findings suggest a number of avenues to improve prevention and treatment of COVID-19 (Table 3).

**Table 3.** Public Health and Health Care Challenges and Opportunities for Improvement

Theme/subtheme	COVID-19 prevention and treatment challenge	Opportunities for improvement
**Theme: COVID-19 was a distant and secondary threat**		
Invincibility	Reduced need to test or protect self from COVID-19	Target Latinx community with Spanish language public health messaging: Use trusted messengers (eg, Latinx physicians/celebrities, community members, spiritual leaders) Integrate low literacy messaging strategies such as use of video and storytelling Treat Latinx youth as distinct subsegment of the broader Latinx community
Misinformation and disbelief	Unclear how to protect against COVID-19	Deliver messages using Latinx-preferred media: Radio, social media, and Spanish television preferred Coordinate outreach via community settings (eg, grocery stores, churches, and schools)^1^ Employ community health workers
Ingrained social norms	Exposure to COVID-19 in group gatherings.	Tailor messages as responsive to social norms: When recommending reduced gatherings, integrate information about how (eg, physical distancing, face masks), where, and when to gather safely
**Theme: COVID-19 was a compounder of disadvantage**		
Fear of unemployment and eviction	Working while symptomatic	Provide financial support and mitigate homelessness: Provide sick leave and financial support for isolation and quarantine Provide unemployment benefits or other economic relief for workers Reduce evictions via economic support and policies Provide loans to Latinx-owned businesses Education about consequences of spreading virus (using above techniques)
	Withholding information about illness from employer, reluctance to be tested	
Lack of safeguards for undocumented immigrants	Reluctance to seek tests or health care	Treat immigrants as integral to any public health response: Identify relief funds/unemployment for undocumented workers Renew 2-year permits for Temporary Protected Status and Deferred Action for Childhood Arrival recipients Address public charge and deportation fears Emphasize importance of influenza (and eventual COVID-19) vaccination
	Presenteeism: working while symptomatic and potentially infecting others because of precarious economic state	
Inability to protect self from COVID-19	Rely on often unsafe public transportation; low access to PPE compound dangers of essential work	Foster safer conditions for those unable to shelter in place: Provide employees and employers with personal protective equipment Enforce physical distance and hygiene in workplace and public transportation Provide personal protective equipment at entrances to public transportation
High-density housing	Spread of infection within households	Provide safe respite to individuals in overcrowded conditions via isolation and quarantine facilities^34^
	Inability to isolate at home	Teach safe practices (using techniques above) including early recognition of symptoms with testing and isolation
**Theme: reluctance to seek medical care**		
Worry about health care costs	Delay in care resulting in increased and possibly more severe COVID-19 infections	Increase access to care regardless of insurance or immigration status: Clarify that emergency Medicaid is not part of the public charge rule change^35,36^ Expand emergency Medicaid to cover outpatient and inpatient COVID-19 care^36^ Expand unemployment health insurance Provide free, low-barrier COVID-19 testing and financial support for individuals who test positive
Concerned about ability to access care if uninsured or undocumented	Individuals that delay care may be more ill once hospitalized	Reduce deportation fear: Explicit public health messaging about the relationship between health care, immigration enforcement, and public charge Work with community-based organizations to increase trust in public health authorities, local health systems, and immigrant communities
Undocumented immigrants fear deportation		
**Theme: health care system interactions**		
Social isolation and change in hospital procedures	Need to address fear and despair during hospitalization	Explicit language-concordant conversation at admission regarding hospital COVID-19 guidelines for care including aspects such as use of phone calls instead of in-person examination by clinician, and limited visitors or hospital workers; if necessary, this can be done by a prerecorded video
Appreciation for clinicians and language access	Language-concordant care facilitates patient-centered care	Maximize language access by embedding language-concordant clinicians or interpreters into COVID care teams; use video interpreters when concordant care not possible
Discharge with insufficient resources or clinical information	Delay in COVID-19 recovery and return to work because of difficulty accessing treatment such as outpatient oxygen	Promote COVID-19 recovery: Expand access to outpatient resources such as oxygen and physical rehabilitation regardless of insurance or immigration status Enhanced social work including links to community wraparound services Include COVID-19 outpatient care as an eligible condition for emergency Medicaid
**Theme: faith and community resiliency**		
Spirituality	Motivating and strengthening patients	Routinely offer spiritual support services when patients are hospitalized
		Provide access to mental health care providers when necessary for associated depression or anxiety
Latinx COVID-19 as advocates	Higher awareness and protection once knowing someone with illness	Engage community members as trusted messengers to promote culture and language-concordant public health messaging within workplaces, schools, and neighborhoods

Abbreviations: COVID-19, coronavirus disease 2019; PPE, personal protective equipment.

A key finding across multiple themes was economic anxiety. The fear of losing wages or becoming unemployed because of COVID-19, coupled with poverty and risk of being evicted, pushed some individuals to work even when symptomatic, thereby contributing to viral spread. Containing COVID-19 infections means mitigating the economic pressures forcing Latinx individuals to work even when infected, regardless of documentation status. One intervention supporting Latinx individuals to isolate when infected, regardless of documentation status, is Right to Recover, a program launched in San Francisco in July 2020 that provided $1285 to workers needing to isolate because of a COVID-19 infection.^37^ The effect of this and similar policies needs evaluation. Individuals also expressed a fear of eviction, despite a moratorium on evictions. Programs that provide rent relief may offer households the economic ability to isolate without fear of unpaid rent.^38,39^

Participants described a reluctance to seek COVID-19 testing and hospital care because of concerns over cost, access to care, and immigration repercussions. A recent analysis among individuals hospitalized with COVID-19 in Colorado found that the median onset between symptom onset and hospitalization was 4 days among Latinx individuals vs 3 days among non-Latinx individuals.^13^ These findings are striking, as Denver and San Francisco both have public hospitals and health care programs that extend care to undocumented individuals at a sliding scale based on income. Both cities also have longstanding sanctuary policies prohibiting the health care system from sharing information with immigration authorities. However, despite these policies, immigration related concerns, including concerns for public charge, remain rampant.^35,36,40,41,42,43,44^

Our study found that Latinx individuals need more and more effective public health messaging to decrease testing fears, improve contact tracing, and encourage symptomatic individuals to seek medical care. Many individuals who recovered expressed the desire to serve as COVID-19 ambassadors within their community. Former patients may be excellent messengers to communicate COVID-19 information and reduce COVID-19 spread. Mobilizing Latinx individuals and community health workers to disseminate culturally specific and language-concordant COVID-19 information may be a powerful strategy to reduce COVID-19 infections (Table 3).^20,45^ Previously hospitalized individuals might also help disseminate COVID-19 vaccine information.^46,47^

The experience of isolation described by participants is common across hospitalized patients with COVID-19.^48,49,50^ However, it may be further exacerbated for Latinx individuals with limited English proficiency and for those with family abroad. To avoid mistrust and confusion, COVID-19–related changes to hospital procedures should be disclosed to patients at admission. For example, Zuckerberg San Francisco General developed an admission Spanish COVID-19 video. It addresses COVID-19 hospital policies and common COVID-19 care practices, such as proning. Technology is also key to linking patients with family members in the US and abroad. Additionally, because participants identified spirituality as important to their recovery, routinely offering spiritual support services to patients could reduce isolation.

Finally, cities and states need policies that improve access to outpatient services at discharge. Outpatient COVID-19 care remains uncovered in many states. This was the case early on in Colorado, and patients paid out of pocket for oxygen or medical care at discharge. Some states, including now Colorado, have included COVID-19 outpatient care as a qualifying condition for emergency Medicaid.^51,52,53^

### Limitations

Our study has limitations. We recruited patients from 2 academic public hospitals in cities where the Latinx population had predominantly immigrated from Mexico and Central America. Transferability of the findings to other health systems or to Latinx immigrants from other regions is unknown. However, as both Denver and San Francisco have extensive public health care systems and a long history of sanctuary policies, the concerns reported by our participants may be even more widespread in other cities. Another limitation is that we only captured the experiences of patients who survived. However, we included individuals who received ICU level care, which captures the experience of those who were severely ill. This study’s strengths include the rich description from participants in 2 cities where Latinx people have faced high COVID-19 infection rates.

## Conclusions

Latinx communities have suffered disproportionately from COVID-19. The pandemic has amplified preexisting inequities in health care and created new disparities in health, economic, and social well-being. Our findings underscore the urgent need for a public health response specific to the Latinx community that harnesses community strengths and offers tailored and specific messages. Results also highlight the need for economic and housing policies that support Latinx individuals’ ability to isolate and protect the safety of essential workers and also the broader community. For “Immigrants (We Get the Job Done)”^54^ to continue to ring true, the US must enact policies to protect workers from COVID-19 and allow them and their household contacts the economic wherewithal to isolate and quarantine.

## Notes

Supplement. **eTable 1.** Interview Guide **eTable 2.** Characteristics of Adult Latinx Survivors Hospitalized for COVID-19 **eFigure.** Thematic Schema by Time Course of Illness
