# Supplementary material for: Experiences of Latinx Individuals Hospitalized for COVID-19: A Qualitative Study
Source: JAMA Netw Open. 2021 Mar 11;4(3):e210684. doi: 10.1001/jamanetworkopen.2021.0684 (PMC7953277; doi:10.1001/jamanetworkopen.2021.0684)
Supplement: Supplement. — eTable 1. Interview Guide eTable 2. Characteristics of Adult Latinx Survivors Hospitalized for COVID-19 eFigure. Thematic Schema by Time Course of Illness [file jamanetwopen-e210684-s001.pdf]

Supplemental Online Content

Cervantes L, Martin M, Frank MG, et al. Experiences of Latinx individuals hospitalized for COVID-19: a qualitative study. *JAMA Netw Open*. 2021;4(3):e210684. doi:10.1001/jamanetworkopen.2021.0684

eTable 1. Interview Guide

eTable 2. Characteristics of Adult Latinx Survivors Hospitalized for COVID-19

eFigure. Thematic Schema by Time Course of Illness

This supplemental material has been provided by the authors to give readers additional information about their work.

© 2021 Cervantes L et al. *JAMA Network Open.*

eTable 1. Interview Guide

1. What was your experience as you were hearing about COVID-19?
    a. What was your source of information?
    b. What were you hearing from others about COVID-19?
2. As you were hearing about some of the changes taking place in your city (such as shelter in place, school closures), what were your concerns and challenges?
    a. Were there things you found helpful during these changes?
3. What measures, if any, did you take to prevent from becoming ill with COVID-19?
    a. Was anything challenging or helpful?
4. When you became ill, did you have any concerns?
5. Once you were hospitalized, what was your experience?
    a. What was challenging or helpful?
6. How was your experience at time of discharge?
    a. Was anything challenging or helpful?
    b. Were you able to follow through with everything that was recommended for you at time of discharge?
7. Following hospital discharge, did you have any challenges or concerns?
    a. What was difficult or helpful during quarantine?
    b. Did this impact your employment?
8. Have you returned to work?
9. Are there any additional challenges or things you have found helpful during the COVID-19 pandemic?

© 2021 Cervantes L et al. *JAMA Network Open.*

eTable 2. Characteristics of Adult Latinx Survivors Hospitalized for COVID-19

Characteristic	DH (n=30)	ZSFG (n=30)	Total (n=60)
**Demographic Characteristics**			
Age, mean (SD),y	49 (11)	46 (14)	48 (12)
Female, No. (%)	14 (47)	10 (33)	24 (40)
Location			
Denver	30 (100)	30 (100)	60 (100)
San Francisco	30 (100)	30 (100)	60 (100)
Preferred interview in Spanish, No. (%)	25 (83)	29 (97)	54 (90)
**Socioeconomic Characteristics**			
Less than high school education, No. (%)	21 (70)	17 (57)	38 (63)
Household income			
<$25,000, No. (%)	16 (57)	15 (50)	12 (38)
$25,000-34,999, No. (%)	5 (18)	7 (23)	12 (20)
$35,000-49,999, No. (%)	5 (18)	4 (13)	9 (15)
$50,000-74,999, No. (%)	2 (7)	4 (13)	6 (10)
Married, No. (%)	10 (33)	13 (43)	23 (38)
Residence in low income area, No. (%)	30 (100)	30 (100)	60 (100)
More than 4 people in home, No. (%)	22 (73)	25 (83)	47 (78)
More than one bathroom in home, No. (%)	14 (47)	9 (30)	23 (38)
More than 2 bedrooms in home, mean (SD)	20 (67)	24 (80)	44 (73)
More than 1 person with COVID-19 at home, No. (%)	23 (77)	27 (90)	50 (83)
Type of work, No. (%)			
Essential Work[a] No. (%)	25 (83)	19 (63)	44 (73)
Critical Trades (construction, electrician, plumbers, etc.), No. (%)	10 (33)	10 (33)	20 (33)
Agriculture/food production (restaurant, animal and crop production), No. (%)	8 (27)	4 (13)	12 (20)
Critical Retail (grocery stores, hardware stores, mechanics), No. (%)	5 (17)	1 (3)	6 (10)
Transportation, No. (%)	0	2 (7)	2 (3)
Healthcare providers (certified nurse and medical assistants), No. (%)	2 (7)	1 (3)	3 (5)
Childcare, No. (%)	0	1 (1)	1 (2)
Other, No. (%)	1 (3)	1 (2)	2 (3)
Unemployed, No. (%)	4 (13)	10 (33)	14 (23)
Among those employed (n=46)	(n=26)	(n=20)	(n=46)
Ability to work from home, No. (%)	2 (8)	2 (10)	4 (9)
Received sick leave, No. (%)	9 (35)	3 (15)	12 (26)
Use of personal protective equipment at work, No. (%)	18 (69)	14 (70)	32 (70)
Employer informed of COVID-19 diagnosis, No. (%)	17 (65)	20 (100)	37 (80)
Lost job due to COVID-19, No. (%)	4 (50)	17 (85)	21 (46)

© 2021 Cervantes L et al. *JAMA Network Open.*

Type of transportation, No. (%)			
Public, No. (%)	6 (20)	14 (47)	20 (33)
Vehicle owner, No. (%)	24 (80)	16 (53)	40 (67)
Insurance type, No. (%)			
DFAP/Healthy San Francisco (program that covers undocumented San Francisco residents), No. (%)	8 (27)	10 (34)	18 (30)
Emergency Medicaid (hospital coverage for undocumented Denver residents), No. (%)	14 (47)	4 (13)	18 (30)
Medicaid, No. (%)	4 (13)	15 (50)	19 (32)
Medicare, No. (%)	2 (7)	0 (0)	2 (3)
Commercial, No. (%)	2 (7)	0 (0)	2 (3)
**Clinical Characteristics**			
Symptoms on admission			
Cough, No. (%)	25 (83)	22 (73)	47 (78)
Shortness of breath, No. (%)	22 (73)	22 (73)	44 (73)
Abdominal pain, No. (%)	7 (23)	5 (17)	12 (20)
Diarrhea, No. (%)	10 (33)	8 (27)	18 (30)
Myalgia, No. (%)	15 (50)	11 (37)	26 (43)
Dysgeusia, No. (%)	4 (13)	0	4 (7)
Anosmia, No. (%)	7 (23)	1 (3)	8 (13)
Flu shot received in the past year, No. (%)	14 (47)	5 (17)	19 (32)
Comorbidities			
Body Mass Index, mean (SD)			
<30	16 (53)	11 (37)	27 (45)
30 to 34.9	6 (20)	12 (40)	18 (30)
35+	8 (27)	7 (23)	15 (25)
Diabetes, No. (%)	10 (33)	13 (43)	23 (38)
Hypertension, No. (%)	10 (33)	8 (27)	18 (30)
Cardiovascular disease, No. (%)	1 (3)	2 (7)	2 (7)
Chronic lung disease, No. (%)	4 (13)	2 (7)	6 (2)
Hospital Course			
Hospital length of stay, mean (SD) days	7 (8)	10 (11)	8 (10)
Intensive care unit stay, No. (%)	5 (17)	12 (40)	17 (28)
Intensive care unit, length of stay, mean (SD) days	14 (11)	12 (10)	17 (28)
Intubated, No. (%)	3 (10)	7 (23)	10 (17)
Acute respiratory distress syndrome, No. (%)	5 (17)	12 (40)	13 (22)
Acute kidney failure, No. (%)[b]	0	3 (10)	3 (5)
Acute liver injury, No. (%)[c]	0	1 (3)	1 (2)
Co-infection with bacterial pneumonia, No. (%)	1 (3)	4 (13)	5 (8)
Disposition			

© 2021 Cervantes L et al. *JAMA Network Open.*

Home, No. (%)	30 (100)	18 (60)	48 (80)
Hotel, No. (%)	0	10 (33)	10 (17)
Other, No. (%)	0	2 (7)	2 (3)
Discharged with oxygen	7 (23)	3 (10)	10 (17)

[a]Essential Work definition[1,2]

[b]Acute kidney injury defined as increase in serum creatinine by 0.3mg/dL or more (>26.5 umol/L) within 48 hours or increase in serum creatinine up to 1.5 times or more baseline within prior 7 days compared to preceding 1 year of data in acute medical records

[c]Acute liver injury defined as an elevation of AST or ALT of more than 15 times the upper limit of normal

1. National Conference of State Legislatures. COVID-19: Essential Workers in the States. 2020; https://www.ncsl.org/research/labor-and-employment/covid-19-essential-workers-in-the-states.aspx#:~:text=All%20workers%20who%20are%20part,Infrastructure%20Security%20Agency%20(CISA). Accessed October 12, 2020.
2. Cybersecurity & Infrastructure Security Agency. Guidance on the Essential Critical Infrastructure Workforce. 2020; https://www.cisa.gov/publication/guidance-essential-critical-infrastructure-workforce.

© 2021 Cervantes L et al. *JAMA Network Open.*

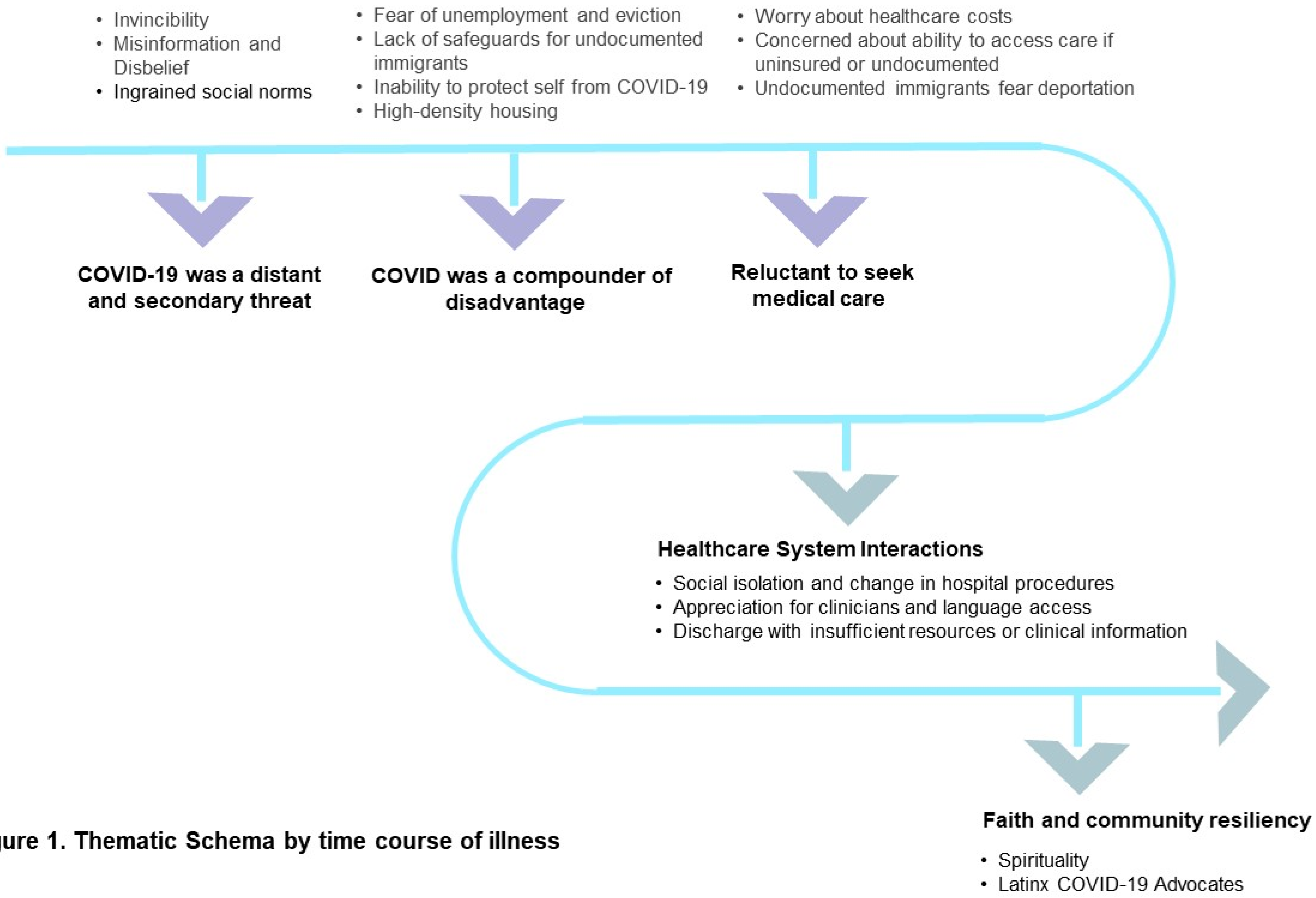

eFigure 1. Thematic Schema by time course of illness

© 2021 Cervantes L et al. *JAMA Network Open.*
